# Protection induced by malaria virus-like particles containing codon-optimized AMA-1 of *Plasmodium berghei*

**DOI:** 10.1186/s12936-019-3017-2

**Published:** 2019-12-03

**Authors:** Dong-Hun Lee, Ki-Back Chu, Hae-Ji Kang, Su-Hwa Lee, Manika Chopra, Hyo-Jick Choi, Eun-Kyung Moon, Kyung-Soo Inn, Fu-Shi Quan

**Affiliations:** 1 Department of Biomedical Science, Graduate School, Kyung Hee University, Seoul, Korea; 2 Department of Chemical and Materials Engineering, University of Alberta, Edmonton, AB T6G 1H9 Canada; 3 Department of Medical Zoology, Kyung Hee University School of Medicine, Seoul, Republic of Korea; 4 Department of Pharmaceutical Science, College of Pharmacy, Kyung Hee University, Seoul, 02447 Republic of Korea; 5 Medical Research Center for Bioreaction to Reactive Oxygen Species and Biomedical Science Institute, School of Medicine, Graduate School, Kyung Hee University, Seoul, Republic of Korea

**Keywords:** *Plasmodium berghei*, Apical membrane antigen 1 (AMA-1), Virus-like particles, Codon-optimized, Vaccine

## Abstract

**Background:**

Despite the extensive endeavours, developing an effective malaria vaccine remains as a great challenge. Apical membrane antigen 1 (AMA-1) located on the merozoite surface of parasites belonging to the genus *Plasmodium* is involved in red blood cell invasion.

**Methods:**

Influenza virus-like particle (VLP) vaccines containing codon-optimized or native (non-codon optimized) AMA-1 from *Plasmodium berghei* were generated. VLP-induced protective immunity was evaluated in a mouse model.

**Results:**

Mice immunized with VLP vaccine containing the codon-optimized AMA-1 elicited higher levels of *P. berghei*-specific IgG and IgG2a antibody responses compared to VLPs containing non-codon optimized AMA-1 before and after challenge infection. Codon-optimized AMA-1 VLP vaccination induced higher levels of CD4^+^ T cells, CD8^+^ T cells, B cells, and germinal centre cell responses compared to non-codon optimized AMA-1 VLPs. Importantly, the codon-optimized AMA-1 VLP vaccination showed lower body weight loss, longer survival and a significant decrease in parasitaemia compared to non-codon optimized VLP vaccination.

**Conclusion:**

Overall, VLP vaccine expressing codon-optimized AMA-1 induced better protective efficacy than VLPs expressing the non-codon optimized AMA-1. Current findings highlight the importance of codon-optimization for vaccine use and its potential involvement in future malaria vaccine design strategies.

## Background

Malaria is a disease caused by *Plasmodium* parasites that may be transmitted to people by female *Anopheles* mosquitoes. An estimated 219 million cases of malaria have been reported in 87 countries in 2017 [1]. The World Health Organization (WHO) estimates over one million deaths by malaria infection each year, which is a severe public health problem [2]. Anti-malarial drugs for human use, indoor spraying with residual insecticides, and the use of insecticide-treated mosquito nets can prevent and reduce malaria transmission. However, 68 countries have reported mosquito resistance to at least one class of insecticide from 2010 to 2017, including 57 reported resistances to 2 or more insecticide classes.

In spite of much effort devoted to develop vaccine against malaria infection, RTS,S/AS01 (RTS,S) is the only vaccine that has shown a protection rate of 26–50%, preventing approximately 4 in 10 cases of malaria [3]. Thus, it is critical to develop more effective vaccines. Malaria vaccines can be aimed to the various stages of antigens of the parasite life cycle, especially the stage that involves host red blood cells (RBCs) [4, 5]. Currently, there are several malaria vaccines under development, with the most promising of them being apical membrane antigen 1 (AMA-1) derived from asexual blood-stage antigens found on the merozoite surface. AMA-1 is a protein of apicomplexan parasites that is essential for host cell invasion [6]. Antibodies against ectodomain of AMA-1 have been shown to prevent RBC invasion and become an important target against *Plasmodium falciparum* blood stage [4]. Although *P. falciparum* AMA-1 polymorphisms remain a major challenge for vaccine development, the authors hypothesized that a virus-like particle (VLP) vaccine expressing AMA-1 would prevent merozoite entry into RBCs and thus play an important role in protection against malaria infection.

The high level of protein expression in heterologous hosts is one of the major bases of modern biotechnology [7]. Multiple strategies regulate and influence gene expression levels, and GenScript OptimumGene^TM^ algorithm takes into consideration as many of them as possible, producing the single gene that can reach the highest possible level of expression. Codon-optimization includes strategies involving gene design engineering, which has a significant impact on gene expression levels and protein folding [7]. Improved expression of the gene could enhance the vaccine efficacy from recombinant protein vaccine. It has also been reported that there is a difference between codon-optimized and native codon in inducing in vitro transfection and T cells expression levels [8]. Although codon-optimization has been shown to improve the expression of human genes in *Escherichia coli* [9], the effect of gene sequence optimization on protein expression using insect cell remains unreported to date. Thus, in the current study, *Plasmodium berghei* AMA-1 sequence was optimized and protective immunity induced by VLPs expressing codon-optimized AMA-1 [AMA-1 (G)] with VLPs expressing the non-codon optimized AMA-1 [AMA-1 (O)] were compared.

In this study, the vaccine efficacies in mice immunized with VLPs containing codon-optimized or non-optimized AMA-1 were evaluated. Immunized mice were challenge-infected with *P. berghei* and protective immunity was assessed. Malaria-specific IgG and IgG2a antibody responses, as well as T cell and B cell responses were induced to a higher extent in the codon-optimized VLPs than the non-codon optimized VLPs. Compared to non-codon optimized VLPs, codon-optimized VLPs also showed less body weight reduction and increased survival rate, as well as significant reduction in parasitaemia in the blood.

## Methods

### Ethics statement

All experimental procedures and animal experiments in this study were reviewed, approved, supervised, and performed in accordance with the guidelines of the Kyung Hee University IACUC (Permission Number: KHUASP (SE)-17-066). Isoflurane anesthesia was used to minimize animal suffering during sacrifice.

### Animal purchase, parasite maintenance and antibodies

Female BALB/c mice were purchased from KOATECH (Pyeongtaek, Gyeonggi-do, Korea), and *P. berghei* (ANKA strain) was maintained in mice by serial intraperitoneal passage. *Spodoptera frugiperda* SF9 cells were maintained in suspension in serum-free SF9 II medium (Invitrogen, Carlsbad, CA, USA) at 27 °C in Erlenmeyer flasks in an incubator set at 120–130 rpm. *Plasmodium berghei*-infected sera from mice were collected through retro-orbital plexus puncture. Horseradish peroxidase (HRP)-conjugated goat anti-mouse immunoglobulins IgG, IgG1, IgG2a and IgG2b were purchased from Southern Biotech (Birmingham, AL, USA). Monoclonal mouse anti-M1 antibody was purchased from Abcam (Cambridge, UK).

### Cloning of *Plasmodium berghei* AMA-1 and influenza M1 genes

For plasmid constructions, AMA-1 gene from the original sequence (accession number: XM_672965.2, 1671 bp) and influenza matrix protein 1 (M1) gene (accession number: EF467824, 1027 bp) were cloned into baculovirus vector pFastBacTM, as described previously [10, 11]. Plasmid containing codon-optimized AMA-1 was synthesized and provided by GenScript (NJ, USA).

### Generation of recombinant baculovirus (rBV)

Recombinant baculoviruses (rBVs) expressing codon-optimized AMA-1, non-codon optimized AMA-1, or influenza M1 were generated as described previously [10, 11]. To generate VLPs, Sf9 cells were co-infected with either the rBV expressing codon-optimized AMA-1 or non-codon optimized AMA-1, together with rBV expressing influenza M1 as a core protein, as conducted previously [8, 12]. Sf9 cell size and proliferation were observed at 5 different areas and photographed under 200× magnification (Leica DMi8). Ten cells from each area were measured for statistical analysis. The produced VLPs were separated from SF9 cells by centrifugation at 4 °C and 6000 rpm for 30 min. Then, VLPs were ultra-centrifuged at 4 °C and 30,000 rpm for 50 min. Pelleted VLPs were purified by a sucrose gradient method by centrifugation at 4 °C and 30,000 rpm for 1 h, and the purified VLPs were pelleted at 4 °C and 30,000 rpm for 1 h as well [10, 11]. VLPs were incubated with phosphate buffered saline (PBS) overnight at 4 °C and protein concentrations were measured using the BCA Assay Kit (Thermo Fisher Scientific, Waltham, Mass., USA). VLPs were stored at − 70 °C until use.

### Characterization of VLPs

Characterization of VLPs was confirmed using Western blot and electron microscopy [11]. VLPs were observed on transmission electron microscopy (TEM) (JEOL 2100, JEOL USA, Inc., Peabody, MA, USA) [13]. Western blots were probed with a primary antibody (anti-*P. berghei* polyclonal antibody) and then a secondary antibody (HRP-conjugated anti-mouse IgG). The M1 protein was detected by a monoclonal mouse anti-M1 antibody. The intensity of AMA-1 in VLPs was calculated by ImageJ.

### Mice immunization and challenge

Seven-week old female BALB/c mice were divided into four groups (n = 10 per group) for non-codon and codon-optimized VLPs comparison experiments. The mice were immunized twice via intramuscular (IM) injections of 100 μg VLPs per mouse. Four weeks after the second immunization, the mice were infected with 1 × 10^5^ of *P. berghei* (ANKA) intraperitoneally (IP). On day 10 post infection, half of the mice in each group (n = 5 per group) were sacrificed and mouse blood and spleen samples were collected. The remaining mice were observed daily for changes in body weight and survival rate, and mice that lost 20% of their body weight were humanely euthanized.

### *Plasmodium berghei*-specific antibody responses

Mice sera were collected from all groups after prime and boost immunization. *Plasmodium berghei*-specific IgG antibodies were determined by enzyme-linked immunosorbent assay (ELISA), and *P. berghei* antigens were prepared as previously described [10, 14]. Blood infected with 25% or more malaria was centrifuged at 6000 rpm at 4 °C for 10 min to separate only RBCs, which was then dissolved in 0.15% saponin at room temperature (RT), and the isolated parasites were washed three times with PBS. The precipitated parasites were sonicated three times at 40 Hz for 30 s and used as an antigen for ELISA. Briefly, a 96-well immunoplate was coated with 2 μg/mL of *P. berghei* antigen and incubated overnight at 4 °C. Then, 100 μL of the serum samples (diluted 1:100, 1:200, 1:200, 1:400, 1:800, 1:1600, 1:3200, 1:6400 in PBST) were added to each well and incubated at 37 °C for 2 h as a primary antibody response. HRP-conjugated goat anti-mouse IgG in PBST (100 μL/well, diluted 1:2000 in PBST) were used to determine *P. berghei*-specific IgG response.

### T cells, B cells, and germinal centre responses by flow cytometry

The population of T cells (CD4^+^ and CD8^+^), B cells, and germinal centres from the splenocytes of mice, 10 days after challenge infection, were analysed by flow cytometry. Briefly, splenocytes (1 × 10^6^ cell/mL) in a staining buffer were incubated for 30 min at 4 °C with Fc Block (clone 2.4G2; BD Biosciences, CA, USA). For surface staining, the surface antibodies (CD3e-PE-Cy5, CD4-FITC, CD8a-PE, B220-FITC, CD19-PE-Cy7, GL7-PE; BD Biosciences, CA, USA) were incubated with the cells for 30 min at 4 °C. The spleen cells were washed with a staining buffer at 4 °C and fixed in ice with 4% paraformaldehyde for 30 min. Then, by using the BD Accuri C6 Flow Cytometer (BD Biosciences, CA, USA), the data was analysed.

### Parasitaemia

To stain infected RBCs, 2 μL of RBCs from the blood of *P. berghei*-infected mice were collected in a tube containing 500 U/mL heparin in PBS. RBCs from *P. berghei*-infected mice were stained using 1 μL SYBR Green I (10,000 × concentrate in DMSO, Cat. No. S9430; Thermo Fisher Scientific, MA, Waltham, USA) [15]. The samples were incubated in the dark at 37 °C for 30 min, and then flow cytometry was performed.

### Statistics

The data was analysed statistically using the One-way ANOVA with Tukey’s post hoc test or student t-test using PC-SAS 9.4 (SAS Institute, Cary, North Carolina, USA). A P value < 0.05 was considered to be significant.

## Results

### Generation of recombinant constructs

**Fig. 1:**
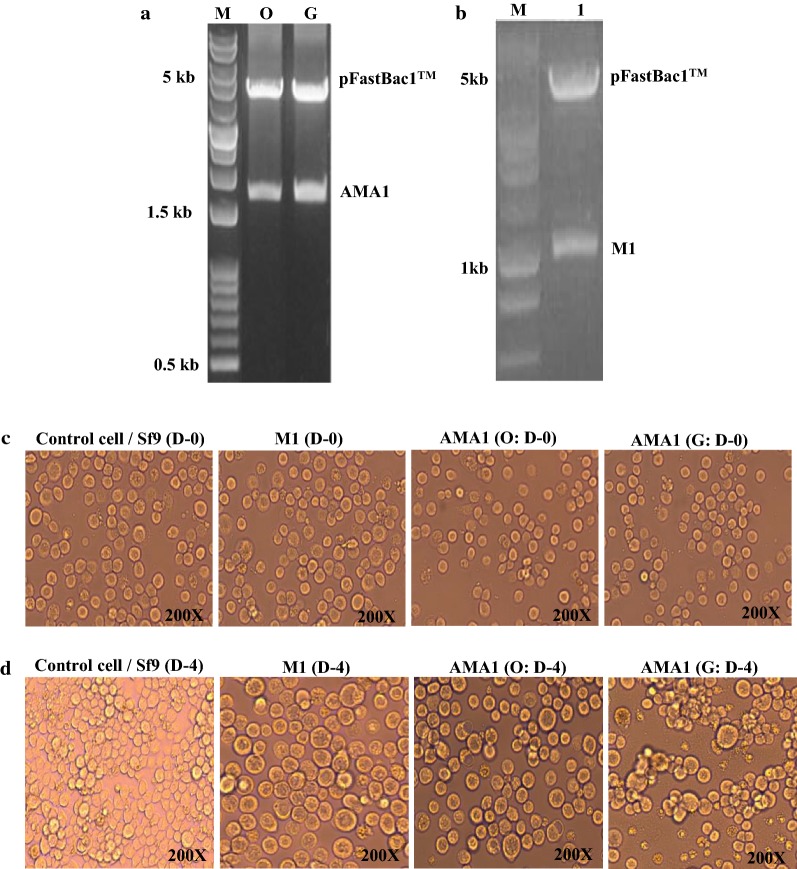
Construction of pFastBac1™ vectors and recombinant baculoviruses (rBV) generation. *Plasmodium berghei* AMA-1 gene and influenza M1 gene were cloned into pFastBac1™ with EcoRI/I and EcoRI/XhoI enzymes, respectively, resulting in *P. berghei* AMA-1 plasmid (**a**) and M1 plasmid (**b**). *M* marker, *O* native(non)-codon optimized gene, *G* codon-optimized gene, *1* M1 gene. Normal SF9 cells (**c**) and recombinant baculoviruses expressing influenza M1, AMA-1 non-codon optimized or codon-optimized gene on day 4 (**d**)

**Fig. 2:**
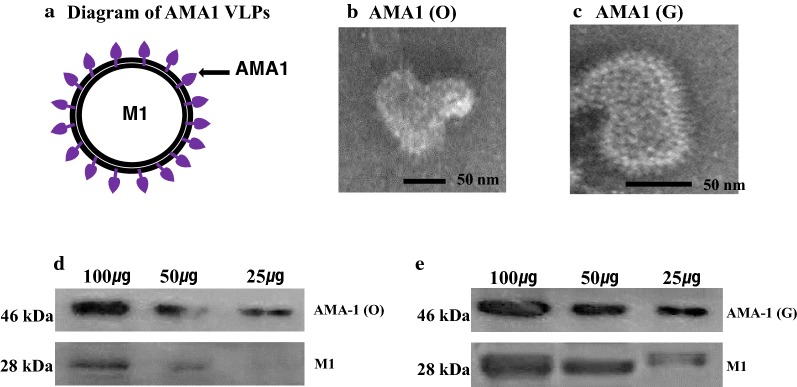
Characterization of virus-like particles (VLPs). Diagram for influenza VLPs displaying *P. berghei* AMA1 (**a**). Electron microscopy of VLPs containing AMA-1 from non-codon optimized (**b**) and codon-optimized VLPs (**c**). Western blot analysis for non-codon optimized and codon-optimized VLPs (**d**, **e**). VLPs (100 μg, 50 μg, 25 μg) were loaded for SDS-PAGE. Polyclonal mouse anti-*P. berghei* antibody was used to probe *P. berghei* AMA-1 protein (1671 bp/46 kDa) and anti-M1 monoclonal antibody was used to determine influenza M1 protein (1027 bp/28 kDa)

As shown in Fig. 1a, b, plasmids pFastBac1™ containing the original AMA1 of *P. berghei* and influenza M1 were PCR-amplified and cloned into pFastBac1™ vectors. As seen in Table 1, codon-optimized AMA-1 was constructed by GenScript, and codon-optimization of a single gene resulted in higher level of protein expression compared to the original AMA-1 (Fig. 2c, d). To determine Sf9 cells infected by baculovirus, Sf9 cells and proliferation were observed under 200× magnification. As seen in Fig. 1c, d, recombinant baculoviruses expressing original, codon-optimized, or influenza M1 in SF9 cells were generated. Baculovirus infected Sf9 cells were found to be significantly larger on day 4 than on day 0, whereas in the naïve control, SF9 cells appeared to maintain the original size and were more highly proliferated on day 4 than those at day 0.

**Table 1:**
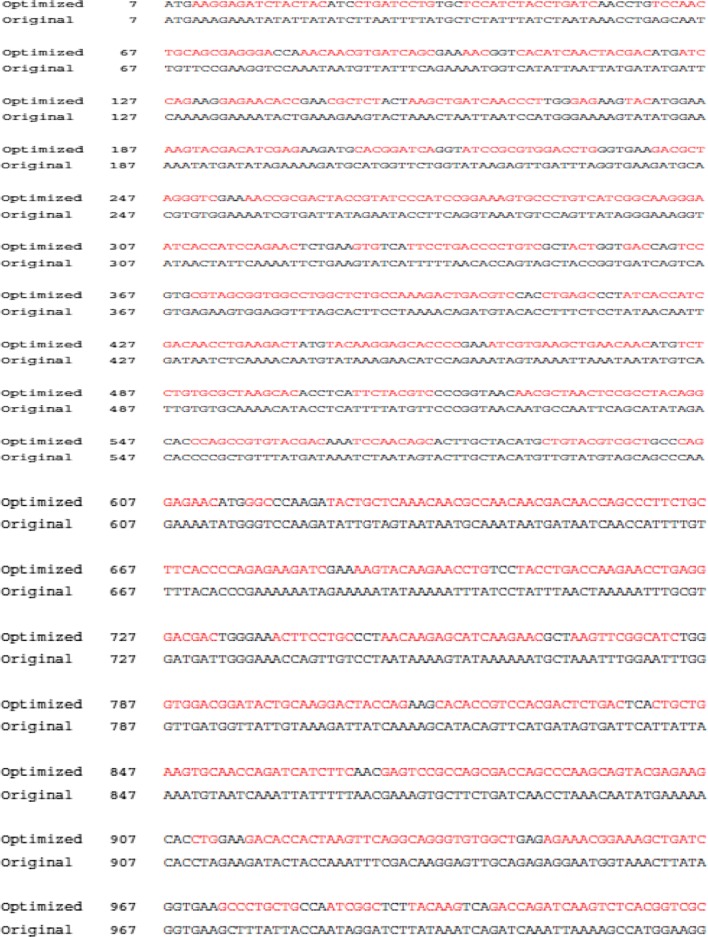

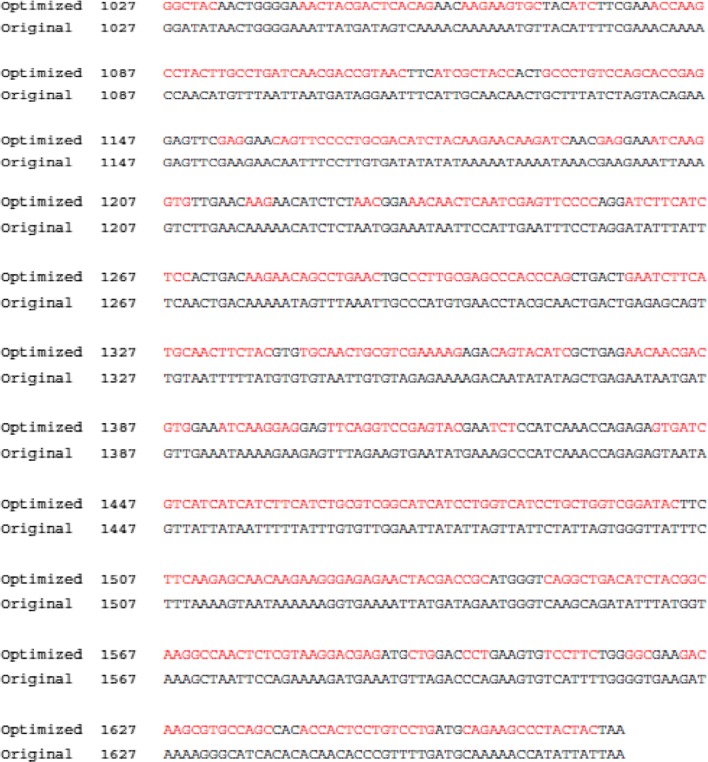
*P. berghei* AMA-1 sequence comparison

Sequences were obtained from the GenScript for codon-optimized AMA-1 and from NCBI sequence database for original non-codon optimized AMA-1 (XM_672965.2)

### Generation and characterization of VLPs

VLPs expressing AMA1 (O) or AMA (G) together with influenza M1 were generated (Fig. 2). Figure 2a is a diagram depicting AMA-1 on the influenza virus VLP surface. When visualized under microscopy, *P. berghei* containing original AMA-1 [AMA-1 (O)] and codon-optimized [AMA1 (G)] VLPs were found to exhibit a spherical shape with spikes on the surface (Fig. 2b, c). Original AMA-1 containing VLPs and codon-optimized VLPs co-infected with M1 were identified by Western blot using *P. berghei* polyclonal antibody and M1 monoclonal antibody. As shown in Fig. 2d, e, codon-optimized VLPs exhibited better protein expression (1.5 times) than original AMA1 VLPs. Proteolytic cleavage has occurred, causing fragmentation of the AMA-1 protein (46 kDa). The full-length AMA-1 proteins at 63 kDa were detected to lesser extent than its fragmented form.

### VLPs immunization elicits *Plasmodium berghei*-specific antibody responses

**Fig. 3:**
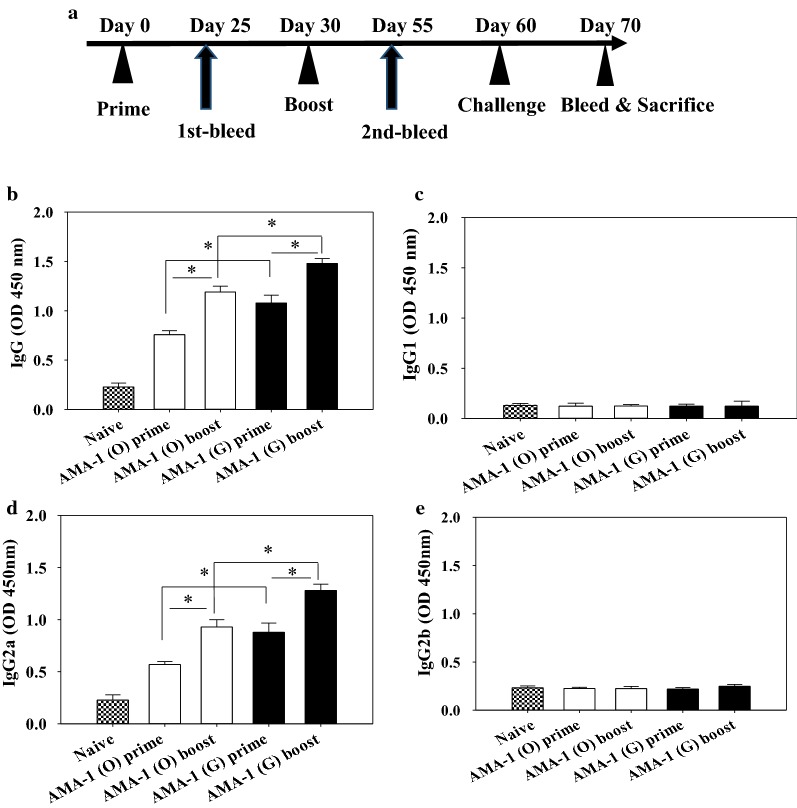
Experimental schedule and *Plasmodium berghei*-specific antibody responses upon immunization. Mice were immunized twice with VLPs, blood was collected, challenge infected and sacrificed as indicated in experimental schedule (**a**). *P. berghei*-specific IgG, IgG1, IgG2a and IgG2b antibody responses in the sera were determined after prime and boost (**b**–**e**, *P < 0.05). Data are expressed as mean ± SD. AMA-1 (O): non-codon optimized VLPs, AMA-1 (G): codon-optimized VLPs

**Fig. 4:**
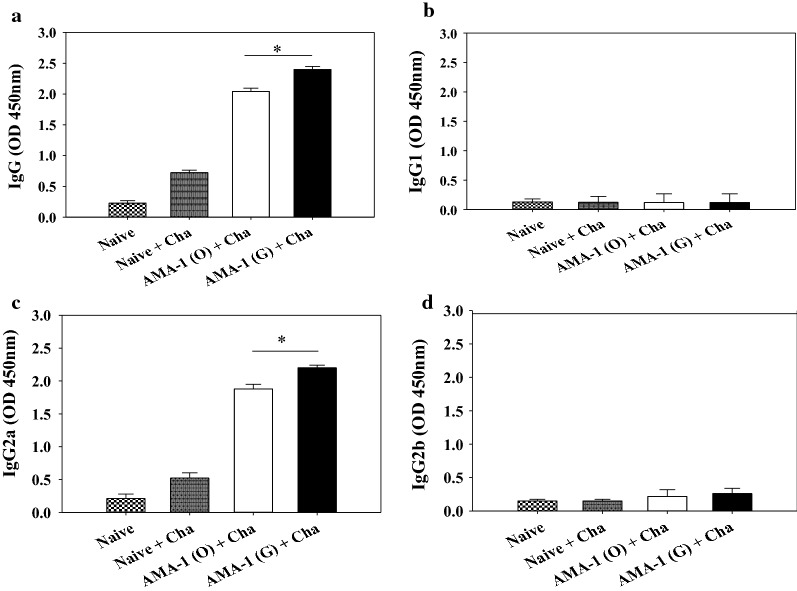
*Plasmodium berghei*-specific antibodies responses upon challenge infection. Immunized mice were challenge-infected (IP) with P*. berghei* at week 4 after boost as indicated in the experimental schedule. *P. berghei*-specific IgG (**a**), IgG1 (**b**), IgG2a (**c**) and IgG2b (**d**) antibody responses in the sera were determined on day 10 post-challenge (**a**–**d** mean ± SD, *P < 0.05). *Cha* challenge infection. Data are expressed as mean ± SD

Mice were immunized with VLPs vaccine and antibody responses were determined. Mice immunized with AMA1 (G) VLPs showed significantly higher levels of *P. berghei*-specific IgG and IgG2a antibody responses in after prime and boost compared to mice immunized with non-codon optimization VLPs (Fig. 3a, b, d; *P < 0.05). However, IgG1 and IgG2b antibody responses were not detected (Fig. 3c, e). Upon challenge infection, codon-optimized VLPs-immunized mice also showed significantly higher levels of *P. berghei*-specific IgG and IgG2a antibody responses than mice immunized with non-codon optimized VLPs (Fig. 4a, c). In addition, IgG1 and IgG2b antibody responses were not detected, as seen before infection (Fig. 4b, d; *P < 0.05).

### VLPs immunization induces T and B cells responses

**Fig. 5:**
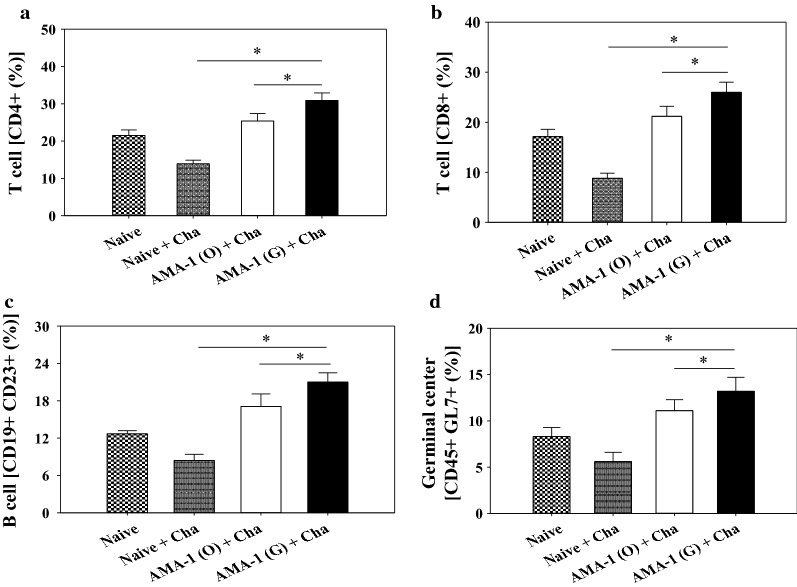
T cells, B cells and germinal centre cell response. Immunized mice were challenge-infected and sacrificed 4 weeks post-challenge as scheduled. CD4^+^ T cells (**a** *P < 0.05) and CD8^+^ T cells (**b** *P < 0.05) populations in the spleen were determined. Mature B cells and germinal centre population were determined in the spleen (**c**, **d** *P < 0.05). Data are expressed as mean ± SD

CD4^+^ T cells and CD8^+^ T cells responses, indicators for assessing immunity induced by VLPs immunization, were determined as scheduled after sacrifice. As shown in Fig. 5, the CD4^+^ T cells (34% and 27%; Fig. 5a, *P < 0.05) and CD8^+^ T cells (29% and 23%; Fig. 5b, *P < 0.05) responses in mice immunized with codon-optimized VLPs and non-codon optimized VLPs were observed. These results indicate that mice immunized with the codon-optimized AMA1 VLPs induced higher T cell responses compared to the original AMA1 VLPs. Similarly, as illustrated in Fig. 5c, d, codon-optimized AMA1 VLPs induced higher levels of B cells responses and germinal centre cell responses compared to original AMA1 VLPs (*P < 0.05). These results indicate that codon-optimized AMA1 induced better humoral immunity than original AMA1 VLPs.

### VLPs immunization induces protection against *Plasmodium berghei* challenge infection

**Fig. 6:**
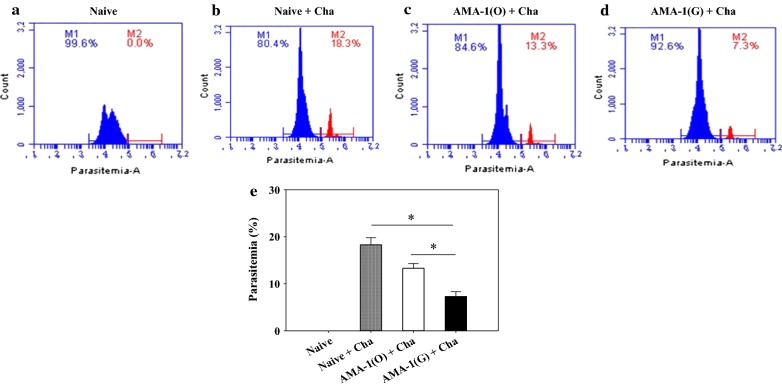
Parasitaemia levels in mice infected with *Plasmodium berghei.* Immunized mice challenge infected with malaria and parasitaemia levels were determined at day 10 post-challenge by flow cytometry. Naïve mice (**a**). Naïve mice infected with *P. berghei*. **b** Mice immunized with VLPs containing AMA-1 non-codon optimized were challenge infected (**c**) and mice immunized with VLPs containing codon-optimized were challenge infected (**d**). The average parasitaemia per group is shown in the bar graph (**e** *P < 0.05)

Parasitaemia in the blood following challenge infection is the most important indicator to assess VLPs vaccine efficacy. Immunized mice and naïve mice were challenge-infected with 1 × 10^5^ *P. berghei* as scheduled (Fig. 6a–d). As seen in Fig. 6e, the immunized mice group significantly reduced parasitaemia in the blood when *P. berghei* infection was attempted. More specifically, the group of mice immunized with codon-optimized VLPs showed the lowest parasitaemia in all groups (Fig. 6d, e, *P < 0.05).

### Immunization with VLPs can increase survival rates against *Plasmodium berghei* challenge infection

**Fig. 7:**
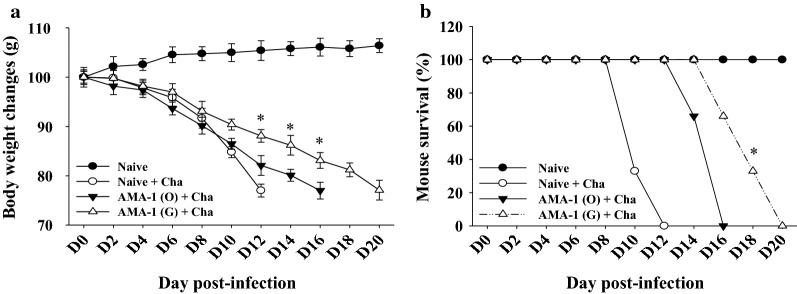
Protection against *Plasmodium berghei* challenge infection. Immunized mice were challenge-infected with 1% lethal dose of *P. berghei* as scheduled at week 4 after boost. For a lethal dose challenge experiment, mice were monitored daily to determine body weight changes (**a**) and survival rate (**b**). Statistical analysis of differences in body weight changes and survival was performed between AMA1 (O) + Cha group and AMA1 (G) + Cha group (* P < 0.05)

Body weight and survival changes were also determined after challenge infections. As shown in Fig. 7, mice immunized with codon-optimized VLPs had a lower weight loss rate than mice immunized with non-codon optimized VLPs (Fig. 7a). Immunized mice with codon-optimized VLPs also survived 4 days longer than those with the original AMA1 VLPs immunization (Fig. 7b), indicating codon-optimized AMA1 VLPs provided better protection than original AMA1 VLPs.

## Discussion

The malaria parasite has a multistage life cycle in which merozoites can invade RBCs, initiating the blood-stage infection of its life cycle. The present study focused on the AMA-1 protein which plays an important role in the merozoite entry into erythrocytes during the blood-stage. This is based on the authors’ hypothesis that immunity by vaccination against AMA-1 could prevent merozoite infection. For this purpose, VLPs were generated using the baculovirus expression system, in which rBV constructs included codon-optimized or non-optimized AMA-1 genes. It was found that the AMA-1 codon-optimized VLPs vaccine significantly reduced parasitaemia than the non-codon optimized VLPs vaccine. This reduction may be attributed to the inhibition of the asexual reproduction of merozoites at the blood stage by inducing higher malaria-specific antibody responses, T cells and B cells responses. These results provide important information on a vaccine design strategy against merozoites in the blood stage by the use of a codon-optimized gene.

Malaria DNA vaccine plasmids, encoded with codon-optimizations of gene fragments of *P. falciparum* merozoite proteins, have been shown to enhance protein expression and immunogenicity in mice [16]. The genes used were malaria EBA-175 RII and MSP-142, in which one in three nucleotides were replaced by different nucleotides to optimize the sequences, and the G+C content was raised by approximately 26–56% in the optimized sequences [16]. It is well-known that the native gene employs tandem rare codons that can reduce the efficiency of translation. In the current study, the codon usage bias has been changed in the insect by upgrading the CAI from 0.64 to 0.96. GC content and unfavorable peaks were optimized from the original AMA-1 gene by GenScript (NJ, USA). After optimization, GC content of AMA-1 reached 53% and the exact same protein sequences were observed in the optimized and original ones. Findings from the current study revealed that AMA-1 (G) VLPs vaccine showed increased immunogenicity and/or vaccine efficacy compared to AMA-1 (O) VLPs, which can be attributed to enhanced AMA-1 expression resulting from codon-optimization. In addition, immunogenicity may have been influenced by differences in the conformation of AMA-1 present in AMA-1 (G) VLPs vs. AMA-1 (O) VLPs. As expected, codon-optimized VLP vaccination enhanced protein expression, resulting in better vaccine efficacy compared to non-codon optimized VLPs.

Antibodies are known to play an important role in controlling blood-stage infections [17, 18]. Mice deficient in mature B cells have shown chronic relapsing parasitaemia, indicating the need for antibodies to control malaria [18, 19]. In the current study, codon-optimized VLP vaccination induced (i) significantly higher levels of mature B cells and germinal centre responses, and IgG and IgG2a antibody responses, and (ii) reduction in parasitaemia when compared to the non-codon optimized VLPs vaccination. These results indicate that antibody response is a critical factor in resisting malaria infection.

As an anti-malarial antibody, IgG2a isotype plays a dominant role in modulating *Plasmodium yoelii* parasitaemia [20]. Murine immunoglobulin IgG1 has been reported not to play a role in the protection against *P. berghei* transgenic model [21]. Consistent with these findings, in the current study, parasite-specific IgG2a antibody responses were detected while no IgG1 antibody responses were found, indicating IgG2a antibody mainly contributes to the significant reduction of parasitaemia.

Malaria infection reveals the exhaustion of parasite-specific CD4^+^ and CD8^+^ T cells, which are mediated by the programmed cell death-1 (PD-1) pathway. Specifically, T cells exhaustion indicates the absence of sterile immunity against blood stage malaria during malaria infection [18]. In this study, unimmunized control mice reduced the CD4^+^ and CD8^+^ T cells responses, whereas VLP vaccination significantly increased CD4^+^ and CD8^+^ T cells responses under both *P. berghei* antigen-stimulated and non-stimulated conditions, indicating better vaccine efficacy induced by VLP immunization. Therefore, the codon-optimized VLP vaccination displayed better protection against malaria by inducing higher levels of CD4^+^ and CD8^+^ T cells responses than the non-codon optimized VLP vaccination.

## Conclusions

Vaccine efficacies were assessed in mice immunized by malaria virus-like particles containing *P. berghei* AMA-1, in which the nucleotide sequence of AMA-1 was codon-optimized or non-codon optimized. Codon-optimized VLP vaccine induced higher levels of parasite specific IgG and IgG2a antibodies, T cells, B cells and germinal centre responses than the non-codon optimized VLPs vaccine. Thus, the codon-optimized VLP vaccine significantly reduced parasitaemia in the blood, showed less body weight loss, and increased the survival rate of mice compared to non-codon optimized VLPs. These results indicate that the codon-optimized VLP vaccine is a promising strategy for the development of an effective vaccine to control the spread of malaria infection.

## Data Availability

Not applicable.
